# PECAplus: statistical analysis of time-dependent regulatory changes in dynamic single-omics and dual-omics experiments

**DOI:** 10.1038/s41540-017-0040-1

**Published:** 2017-12-19

**Authors:** Guoshou Teo, Yun Bin Zhang, Christine Vogel, Hyungwon Choi

**Affiliations:** 1 Center for Genomics and Systems Biology, Department of Biology, New York University, New York, NY USA; 2 College of Arts and Science, New York University, New York, NY USA; 3 Saw Swee Hock School of Public Health, National University of Singapore, Singapore, Singapore; 4 Institute of Molecular and Cell Biology, Agency for Science, Technology, and Research, Singapore, Singapore

## Abstract

Simultaneous dynamic profiling of mRNA and protein expression is increasingly popular, and there is a critical need for algorithms to identify regulatory layers and time dependency of gene expression. A group of scientists from United States and Singapore present PECAplus, a comprehensive set of statistical analysis tools to address this challenge. Protein expression control analysis (PECA) computes the probability scores for change in mRNA and protein-level regulatory parameters at each time point, deconvoluting gene expression regulation in the presence of measurement noise. PECAplus adapted PECA’s mass action model to a variety of proteomic data including pulsed SILAC and generic protein expression data. It also features analysis modules to fit smooth curves on rugged time series observations, and to facilitate time-dependent interpretation of the data for genes and biological functions.  They demonstrate the core modules with two time course datasets of mammalian cells responding to unfolded proteins and pathogens.

## Introduction

Simultaneous, time-resolved profiling of mRNAs and proteins has developed into a routine task, providing new insights into the dynamics of cellular gene expression regulation.^1^ Current next generation sequencing technologies enable whole transcriptome profiling robustly; and mass spectrometry-based proteomics has matured with the ability to quantify several thousands of proteins in complex biological matrices, such as human tissues. Pairing these technologies, emerging studies have provided intriguing insights into the relative contribution of RNA and protein level regulation in response to various types of stress,^2–4^ others have compared ribosome profiling and protein synthesis rates in dynamic conditions.^5^

These two-layered, time-resolved datasets bring new challenges to data analysis, as traditional fold-change and significance analyses methods cannot be used. Currently, the datasets are typically analyzed assuming that a single, fixed first-order ordinary differential equation (ODE) can explain the variation of a gene *across the entire time course*. The ODE equations often take the form of $\frac{{\rm d}}{{{\rm d}t}}Y_t = \kappa _{\rm s}X_t - \kappa _{\rm d}Y_t$, where *Y*_*t*_ and *X*_*t*_ denote protein and mRNA expression levels at time *t*, respectively. The two major kinetic parameters include synthesis rate *κ*_s_ and degradation rate *κ*_d_ and they determine the changes in protein expression given mRNA expression information.^2,6^

However, the ODE-based approach has several limitations when applied to dynamic experiments. First, it implies that the rates of translation and protein degradation remain constant over the entire time period or change linearly at best, which is unlikely to hold true in a rapidly changing cellular environment with long follow-ups. As a result, the method reports only *one* set of rates for each gene. Second, the true nature of the gene expression function, i.e. the relationship between the input and the output, is difficult to recognize in the presence of measurement errors and other sources of noise, especially with a small number of observation time points. Third, the approach is usually unable to deconvolute the contributions of the different regulatory layers, i.e. that of synthesis and degradation, and that of RNA-level and protein-level regulation.

Last but not least, it needs to handle different types of proteomic data, e.g. data from pulsed SILAC experiments^7^ or the protein expression data acquired with label-free, conventional stable isotope labeling-based (e.g., SILAC^8^), or isobaric tagging-based quantification methods (e.g., iTRAQ,^9^ TMT^10^). The challenge with the latter data is often overlooked: without pulsed labeling, it is impossible to distinguish between newly synthesized and pre-existing proteins. To the best of our knowledge, there exists no computational tool that is able to infer rate parameters under the relaxed constraint and identify both significantly regulated genes *and* significant change points in a multi-layered regulatory system.

To address this challenge, we present PECAplus, an ensemble of statistical models for probabilistic inference of single-level or multi-level regulatory kinetic parameters, including direct estimation of synthesis and degradation rates from a variety of datasets. In particular, all models in PECAplus identify *the time point in which the rate parameters shifted*, reporting a statistical significance score called the *change-point probability score* (CPS) for each gene at each time point. We illustrate the models for paired protein–RNA time series data, but they can also be readily fit onto mRNA data alone for the inference of RNA-level regulatory parameters without software modification. PECAplus is based on the core protein expression control analysis (PECA) model,^11^ termed PECA Core hereafter, which uses a regression-like framework for detecting significant changes in the combined effects of synthesis and degradation for individual genes. The underlying model uses a linear cumulative sum equation mimicking an ODE in a time interval manner, which is written as $\Delta E[Y_{t + 1}] = \Delta h_t(\kappa _{{\rm s}t}X_t - \kappa _{{\rm d}t}E[Y_t])$, where the symbol *E*[*Y*_*t*_] denotes denoised (true) protein concentration at time *t* conditional on the observed mRNA concentrations.

**Fig. 1:**
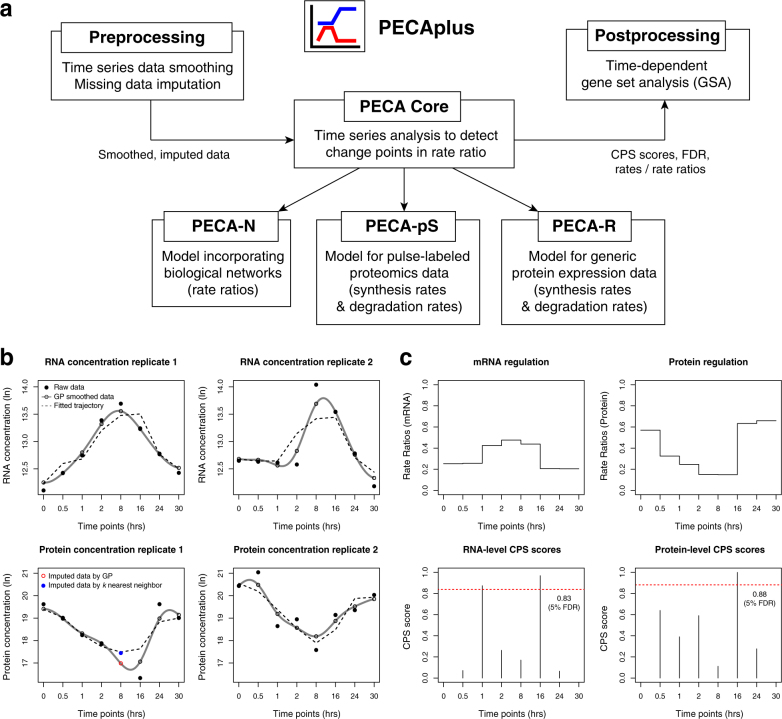
**a** Schematic diagram of PECAplus modules. The pre-processing module performs data smoothing and missing data imputation. The processed data goes through a mass action modeling module of user’s choice, and post-processing GSA module is applied to summarize time-dependent regulation patterns for biological functions. **b** PECA core analysis input and output in SLC39A14 gene. The four panels show the RNA and protein expression data, with solid dots and clear circles representing observed and GP-smoothed data points, respectively. Red circle and blue solid circle are imputed protein expression value at 16 h by GP and *k*-nearest neighbor imputation method. Fitted trajectory is the consensus time course profile across the two replicates reported from the PECA model. **c** The panels on the right side show the inferred rate ratios and CPS values for RNA-level and protein-level regulation. Red dashed lines are the CPS thresholds at 5% FDR (0.83 for RNA, 0.88 for protein)

The analysis using PECAplus occurs in three steps (Fig. 1a): the data pre-processing module applies an advanced curve fitting technique to noisy time series data, resulting in smooth time series for each gene; an analysis module implementing a proper mathematical model for the type of quantitative proteomic data and the goal of the analysis, e.g., rate ratio change point detection or synthesis and degradation rate estimation; and finally the gene set analysis (GSA) module that summarizes the regulatory changes at the level of biological functions in a time-dependent manner.

We demonstrate the different modes of analysis along with the newly implemented pre-processing and post-processing functionalities, using a label-free proteomics and transcriptomics dataset for the unfolded protein response,^4^ and a dataset derived from a pulsed-SILAC experiment paired with transcriptomic data for LPS stimulation.^3^ PECAplus is freely available as a compendium of scripts and as a plugin for the widely used proteomics analysis software PERSEUS.^12^

## Results

### PECA Core: basic approach

PECA Core performs statistical inference on the ratio of protein synthesis rate over degradation rate in individual genes across time points, i.e. for *T*−1 intervals in a *T* time point experiment (rate ratios hereafter). By definition, a change in rate ratio indicates that the balance between synthesis and degradation tips to one direction (up or down), i.e. implicitly assuming that this change is the result of cellular regulation. However, it cannot inform whether the change is due to adjustment of synthesis rate or degradation rate, or both. In particular, PECA Core calculates the probability that the rate ratio is significantly different between adjacent time intervals before and after each time point. We validated and confirmed performance of the core approach in detail in Teo et al.^11^

We first illustrate PECA Core using a paired proteomics and transcriptomics dataset collected from mammalian cells responding to stress of the ER at eight time points.^4^ PECA Core identifies change points of protein-level (i.e. translation/protein degradation) and RNA-level (i.e. transcription/mRNA degradation) regulation. At the protein level, we paired protein expression data with respective RNA expression data. At the RNA-level, we paired RNA expression data with constant values for DNA copy number.^4^

Figure 1b, c show the two-layered regulation output from the PECA Core for ZIP14 (SLC39A14), a zinc transporter with links to ER stress.^13,14^ Under stress, its mRNA expression increases to peak at eight hours, while its protein expression is at a minimum level at that time point. Even if we take into account the typical time delay associated with translation, these opposing expression changes suggest complex interplay between the two levels of regulation, especially considering that the latter four time points are spaced 6–8 h apart between adjacent observation times. Indeed, PECA Core identifies significant RNA rate ratio changes between the 1 and 16 h marks and protein rate ratio changes at the 16 h mark (Fig. 1c), with high protein-level CPS scores near 1 (false discovery rate, or FDR < 0.05). Hence PECA’s change point analysis framework translates the simultaneous time course mRNA–protein data into biologically interpretable measures of mRNA-level and protein-level regulation (rate ratios, upper panels of Fig. 1c), each with associated time-specific statistical significance scores (CPS scores, lower panels of Fig. 1c).

### Gaussian Process (GP) model for data smoothing and imputation

In PECAplus, we introduce a new data pre-processing module which smoothes the typically rugged expression data and imputes missing values based on temporal correlation in the time series setting. This pre-processing is beneficial not only because RNA and protein measurements are intrinsically noisy, but also because this noise can create false time series trends when the number of time points is small.

The module fits a smooth curve on the time series measurements of each gene using a stochastic model called GP. The empty circles connected through the solid lines in Fig. 1b illustrate the smoothing for ZIP14. The GP model has two kernel parameters controlling the smoothness, and we have optimized them with several, representative experimental data sets. Supplementary Information describes the tuning parameters and their impact on smoothed curves. However, we still recommend the user to visually inspect the fitted data using the script included in the package and tune the parameter if necessary as every dataset has different properties, such as varying noise levels.

In addition to smoothing, GP also interpolates *unobserved* time points as the model provides both the estimate at any time point and the uncertainty underlying the prediction. The lower left panel in Fig. 1b illustrates this interpolation of protein measurement in replicate 1 at 8 h (red circle). The imputed value not only removes the ruggedness in the data, but also produces more similar temporal patterns between the replicates. The blue solid circle is the value that would have been imputed by the *k*-nearest neighbor imputation,^15^ which does not model the temporal correlation explicitly and therefore produces sub-optimal imputations. Supplementary Information describes performance evaluation.

### Gene set analysis: time-dependent function enrichment analysis

**Fig. 2:**
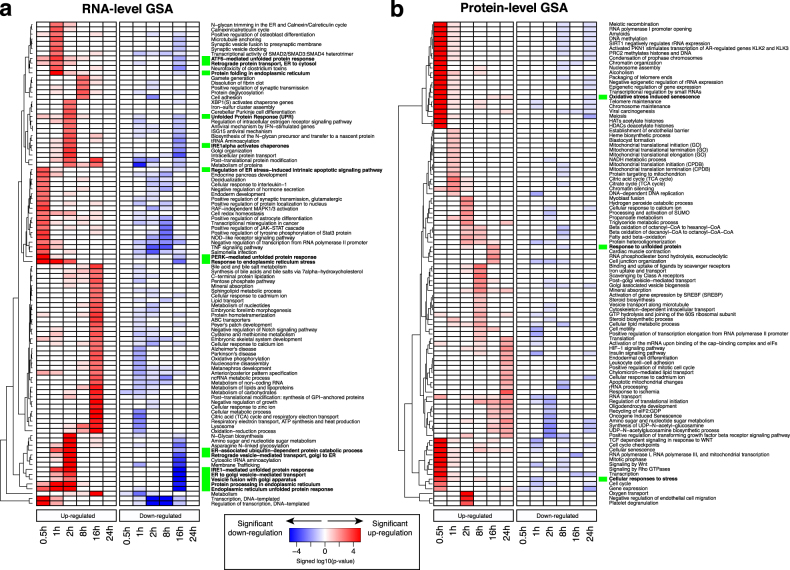
GSA heatmap showing the statistical significance of enrichment of each function in (**a**) RNA-level analysis and (**b**) protein-level analysis. Each cell of the heatmap represents −log_10_ *p*-values times the sign of change, i.e. + for up-regulation and − for down-regulation. Red and blue colors represent the enrichment of pathways in up-regulation and down-regulation at the two levels at each time point. Green squares indicate ER-stress-related biological functions

The large number of gene-level CPS scores and rate ratio parameters reported by PECA for each time point or interval can make it difficult to grasp the overall regulatory dynamics. For this reason, PECAplus offers the GSA module to convert the gene-level output into a summary of significant changes for gene function groups, i.e. all genes annotated with a specific function (Fig. 2). The GSA module performs hypergeometric tests for the enrichment of the Gene Ontology^16^ (GO) terms and other pathways curated in the Consensus Pathway DataBase^17^ (CPDB) in the genes with CPS score above a user specified threshold at each time point (guided by false discovery rate estimates). The test evaluates genes with increased and decreased rate ratios separately, i.e. the different directions of change, and genes with rate ratios altered in both directions, extracting regulatory changes in each biological pathway.

Figure 2 shows the output from the GSA module for ER-stressed mammalian cells, with CPS score thresholds associated with 5% FDR in each analysis. The heatmap shows −log_10_ *p*-values of the most significantly enriched, non-redundant pathways, in a time-dependent manner. It illustrates the dynamic up-regulation and down-regulation of each pathway, at both the mRNA (Fig. 2a) and protein levels (Fig. 2b). We clearly see upregulated pathways implicated in unfolded protein response, ER-associated protein degradation (before 2 h) and various metabolic pathways (16 h) at the mRNA level. In comparison, genes of cell cycle and chromatin organization (30 min), metabolism (1 h), and translation (after 2 h) are upregulated at the protein level.

### PECA-N: Incorporating prior network information in inference

Next, we built the PECA-N module which boosts sensitivity and specificity of the PECA Core approach using prior information from biological networks. The PECA-N module uses a Bayesian inference framework called Markov random field (MRF) prior^18,19^ in cases where connected genes are regulated in a similar fashion, i.e. change their rate ratios along the time course concordantly. The user can supply any network data, e.g. on functional similarity or physical protein–protein interactions. PECA-N then increases sub-threshold scores above the threshold if a gene’s network neighbors are regulated similarly at the same time point. Importantly, if a gene’s network neighbors are regulated inconsistently, then PECA-N will not falsely incorporate the prior to report more changes. In other words, PECA-N increases true-positives without introducing a large number of false-positives.

**Fig. 3:**
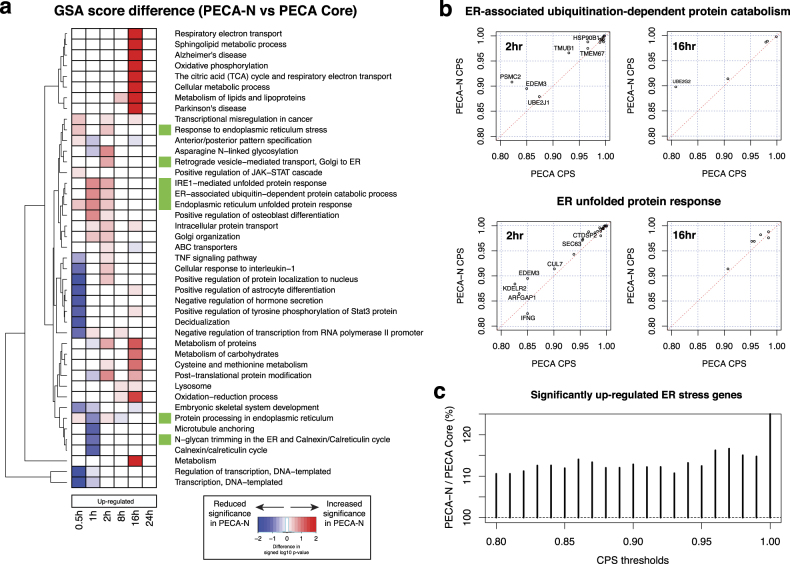
**a** The difference in −log_10_ *p*-values of biological functions between PECA-N and PECA Core for the RNA-level up-regulation. Green boxes indicate ER stress-related biological functions. **b** CPS scores for the genes belonging to ER-associated ubiquitination-dependent protein catabolic process and ER unfolded protein response, for which the PECA-N analysis yielded more significant *p*-values in the GSA module at 2 h. **c** The ratio of the number of significantly up-regulated genes (PECA-N/PECA Core) in the RNA level at the same CPS score thresholds among 296 genes in the ER stress-related biological processes

To demonstrate PECA-N, we first used the protein–protein interaction information from the STRING database^20^ on the RNA-level data of the ER stress experiment. Figure 3a shows the impact of network information with respect to function enrichment between PECA-N and PECA Core using the GSA output. PECA-N made the most notable difference in the GSA scores for upregulated metabolic functions at 16 h and ER stress-related functions at 1 and 2 h. Consistently, the CPS scores for the genes in these two function categories were also elevated in the PECA-N output (Fig. 3b, Supplementary Fig. 1). Treating up-regulation of ER stress-related genes as true positives, Fig. 3c suggests that PECA-N with the STRING network detected 10–15% more genes with up-regulated rate ratios than PECA Core. The CPS values for genes not involved in relevant function categories remained unchanged or became even lower, as reflected by the reduction in the GSA scores of biological processes not related to ER stress (blue cells in Fig. 3a).

Next, we applied PECA-N to the protein-level analysis with the same network information. Interestingly, the network information in PECA-N made little changes to the CPS scores from PECA Core in the protein-level analysis. This mainly suggests that the protein–protein interaction network is better aligned with RNA-level gene expression regulation during ER stress than the protein-level regulation. However, another main reason is the poor network coverage over the 2130 genes in the protein data, which accounts for only 14.6% of the original network. By contrast, the RNA data has ~16,000 genes and >11,000 of those genes appeared on the network, accounting for 67.5% of the network. Thus, the power to detect *additional* coordinated synthesis and degradation change was limited in the protein level analysis of PECA-N. Furthermore, it is possible that protein synthesis and degradation are slow in nature and thus span multiple time periods with varying lengths of time lag, which cannot be captured efficiently by MRF prior structure. Nevertheless, translation control is still highly coordinated over time as the GSA output suggests (Fig. 2b).

### PECA-pS: estimation of rate parameters from pulsed SILAC proteomics data

Next, we developed PECA-pS to parse pulsed SILAC data that allows for quantification of newly synthesized proteins and monitoring of degradation for existing protein copies simultaneously.^7^ Importantly, PECA-pS evaluates each time point separately to account for non-linear changes in rates. We tested PECA-pS against an existing approach that analyzed dendritic cells following LPS treatment.^3^ The authors estimated *per mRNA* protein synthesis rates and degradation rates for 3147 genes, using two different isotope labels for the two rates, respectively (e.g., heavy and medium stable isotopes), and a third channel (light) as reference. The authors then used an ODE model to estimate the rate parameters, but assumed that the rates were linearly increasing or decreasing (or not changing). In contrast to PECA-pS, the approach produced only *one* set of rate estimates for each gene at 0 h and another set at 12 h.

To allow for flexible rate changes, PECA-pS estimates rates *per time interval*. We note that the rate parameters cannot be computed in the absolute molar concentration scale, since most proteomics data sets do not have absolute quantification. Similar to PECA Core, PECA-pS reports CPS scores for a change in the rates between consecutive intervals. An important condition when modeling pulsed SILAC data is that the time course pattern must be monotone decreasing in the channel representing degradation of existing proteins, and monotone increasing in the channel representing synthesis of new proteins. Therefore, we focused the analysis on those proteins where this condition held true (see Online Methods).

**Fig. 4:**
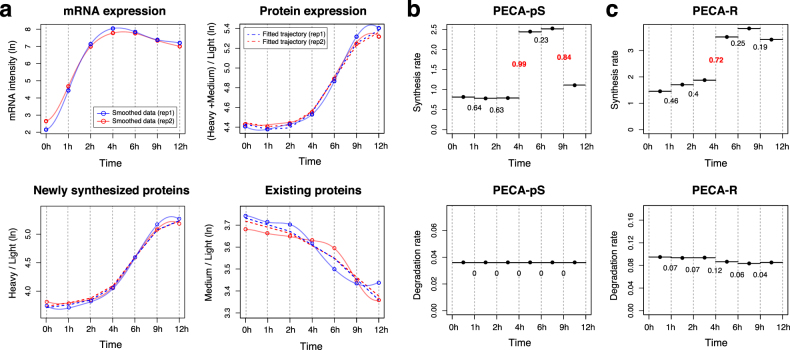
**a** Smoothed data (solid opaque lines) and consensus time course profile inferred by the fitted PECA models (dashed lines, fitted trajectory) for the heavy-labeled and medium-labeled channels for IFIT1 gene in the synthetic data derived from the LPS data set. **b** Synthesis rate and degradation rate profiles, inferred from H/L and M/L channels, respectively, with CPS scores from PECA-pS. **c** Synthesis and degradation rate profiles, simultaneously inferred from (H + M)/L with CPS scores from PECA-R

Figure 4a shows the GP smoothed data (solid line) and PECA-pS fitted time course patterns (dashed line) for the heavy and medium isotope-labeled channels for the IFIT1 gene. As shown in the plot, the model fits a monotone increasing expression profile for the heavy channel as the intensity values in this channel quantify newly synthesized protein copies. We then compared the PECA-pS output to the 0 and 12 h rate estimates from the ODE model used in the original paper,^3^ and observed good correlation confirming our model (avg. *R* ^2^ of 0.48, Supplementary Fig. 2). In addition, PECA-pS reported a high CPS score at 4 h in the synthesis rates.

In sum, PECA-pS is a tool to estimate synthesis and degradation rates from pulsed SILAC-based ratio information, for individual time intervals. While the average PECA-pS rate parameter values over time periods can be interpreted similarly to those from linearly shifting ODE-based model, delivering overall rate estimates, PECA-pS advances the analysis through detection of significant rate changes *for each time point* separately, entirely *independently of the underlying rate function* (Fig. 4b).

### PECA-R: estimation of rate parameters from generic expression data

Last, we present another important PECAplus module to approximate synthesis and degradation rates from paired mRNA and proteomics data, in the absence of pulsed SILAC data (PECA-R). PECA-R can use any type of protein expression values, e.g. concentrations or intensity values. With the rise of label-free proteomics experiments and the increasing use of post-hoc labels, such data becomes more routinely available. We illustrate PECA-R with the LPS data in which we summed medium and heavy channels for each gene to produce total protein expression values.

De-convoluting synthesis and degradation rates from the total protein expression data (not pulse labeled) requires strong mathematical assumptions as the data does not separate newly synthesized and existing molecules. Any change in the concentration of a molecule can be explained by infinitely many combinations of synthesis and degradation rates. Moreover, synthesis and degradation for a gene might have opposing effects and the resulting expression data would be unchanged. Therefore, it is impossible to recover change unless additional information is available, i.e. changing RNA concentrations that impact protein levels.

PECA-R aims to overcome this *identifiability issue* by placing reasonable restrictions on the rate parameter space. Specifically, we assume that increase in total expression of a protein is more likely attributable to increased synthesis than decreased degradation; whereas decrease in expression is more likely due to increased degradation than decreased synthesis (see Online Methods).

**Fig. 5:**
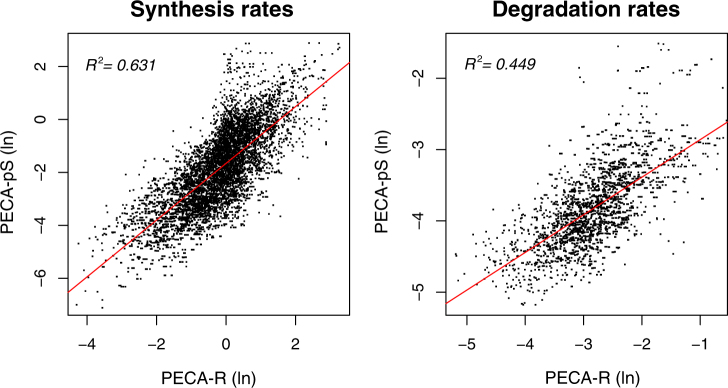
Comparison of rate parameter estimates between PECA-pS and PECA-R in the synthetic data set. Note that each dot is a rate parameter for one time period, not the entire time course. The rates are not in an absolute scale since the mRNA and protein measurements are not made in molar concentrations

To evaluate the ability of PECA-R, we created an unbiased, synthetic data set from the LPS data mirroring data parameters (Online Methods), consisting of 1231 genes. Fig. 5 shows that PECA-R’s approach successfully models the data: the rate estimates from PECA-R in the LPS data set correlate well with the estimates from PECA-pS (with average *R* ^2^ of 0.54). While the rate estimates from the two approaches are not on the same scale, the relative changes within individual genes are well preserved between the two versions (Fig. 5).

Despite similarity in the synthesis rates between the PECA-pS and PECA-R, a number of synthesis rate changes with high CPS scores were specific to the PECA-pS output (Fig. 4c). We found that PECA-R did not detect changes in these genes as their total protein (and RNA) expression values remained largely constant over time (e.g., within 1.5 fold). In contrast, PECA-R sensitively detected rate changes when the total concentration changed substantially due to regulated synthesis or degradation.

Finally, we also validated the PECA-R with the rate values reported by the ODE-based approach with the linearity assumption.^3^ The correlation between the two sets of estimates was very strong (Supplementary Fig. 3, average *R* ^2^ 0.31), supporting the ability of PECA-R to recover the underlying synthesis and degradation rates on a relative scale. Remaining differences between the two approaches can be explained by the fact that many rate parameters changed in a non-linear fashion (Fig. 4b, c), similar to what we observed when comparing PECA-pS and the ODE-based approach.

### Computation time and tool availability

The source code and binaries are freely available from https://github.com/PECAplus. PECAplus is also available as a plugin to the widely used Perseus software (version 1.6.0.2), downloadable from the same GitHub site. The Online Methods describe the availability and computation requirements.

## Discussion

In this work, we presented a comprehensive statistics package to analyze time series omics data that involves one-layer or two-layer expression data. We present PECAplus through two proteomics–transcriptomics examples, but the approach is generalizable to any paired expression data with two levels of regulation, i.e. where the molecules in one level serve as template for synthesis of those in the other level. For example, the researcher might investigate changes in transcription and RNA degradation, using transcriptomics and genomic data. In principle, PECAplus can also be used with paired ribosome footprinting and transcriptomics time series data, in which the tool deconvolutes the contributions of ribosome association with and dissociation from the RNA to support translation (*not shown*).

The main objective of PECAplus is robust inference of gene expression regulatory parameters in a dynamic experimental design, moving beyond traditional fold change analysis that is not suitable for time series data. The most notable advances in PECAplus are PECA-R and PECA-pS, which infer (relatively scaled) rates of synthesis and degradation. Pulsed SILAC experiments monitor these rates directly through assessment of newly made and pre-existing proteins. However, traditional analyses only determine rates across the *entire* time course and ignore rate changes during the experiment. PECA-pS takes the analysis further and infers rates that are specific to each measurement interval, monitoring complex regulatory patterns over time.

Inferring change points of rate parameters directly from proteomics data that was not collected in a pulse-chase experiment can be a risky endeavor. We strongly recommend first analysis of such data to be carried out with PECA Core or PECA-N to identify genes, gene groups, and time points with significant changes. The user can perform post hoc analysis using PECA-R to identify the possible *cause* of the change, i.e. differentiate between synthesis-driven or degradation-driven events. If the proteomics data is from a pulsed SILAC experiment, PECA-pS can extract rate parameter changes more accurately than PECA-R to determine to estimate synthesis or degradation rates. Therefore, we recommend using PECA-pS over PECA-R in this case. When the experimental design does not include pulse labeling, we recommend using PECA-R to examine rate parameters, but strictly focus events with high CPS scores associated with noticeable and statistically significant impact on the total protein concentration changes.

In sum, PECAplus offers an array of solutions to decipher systems-level signals from data generated with different experimental platforms. It employs mathematically sound statistical analysis of paired omics time series data in stream-lined fashion. In contrast to traditional analysis of concentration changes, PECAplus generates hypotheses on the regulatory mechanism underlying the change, e.g. if it arose from synthesis or degradation of the molecule. It helps moving gene expression analysis to new levels: that of time and of interconnected regulatory layers.

## Methods

### ER stress data

We used the whole transcriptome RNA-seq data and proteomic data from Cheng et al.^4^ for 2131 genes with missing observations at *up to two time points* within each replicate. The experiment consists of mRNA and protein intensity data collected at eight time points (0, 0.5, 1, 2, 8, 16, 24, and 30 h) in two biological replicates of HeLa cells after DTT treatment. This data set was used for illustration of data smoothing and imputation, and time-dependent functional enrichment analysis in PECA and PECA-N analyses.

### LPS data

We obtained the pulse labeled-intensity data for 2288 genes from supplementary data in Jovanovic et al.^3^ Using a modified pulsed-SILAC strategy,^7^ the abundance of newly synthesized proteins (heavy isotope-labeled, H) and previously labeled proteins (medium isotope-labeled, M) are measured up to 12 h after LPS treatment on dendritic cells. We divided the intensity values into the medium-isotope and heavy-isotope labeled samples by those in the light-labeled samples (H) to adjust for the variation in the reference pool of dendritic cells. This data was used for the illustration of PECA-pS.

### Synthetic data derived from the LPS data set

To evaluate PECA-R, we derived a synthetic data set from the original LPS data by summing the intensity data from the medium-labellled and heavy-labeled channels at each time point (separately within each biological replicate), in addition to normalization by light-labeled samples at respective time points. The original data demonstrated many time course patterns with abundance values defying the expected trajectories in some genes: the intensity values of newly synthesized proteins decreased over time, or the intensity values of existing proteins increased in some genes. We removed these genes to avoid complications in the evaluation. We further smoothed both channels by fitting PECA-pS to guarantee generally smooth, monotone decreasing or increasing curves in the original signal, and added random noise to the filtered data (Gaussian noise, mean 0, standard deviation 0.1). This new data set consisting of 1231 genes was used for the illustration of PECA-R and the comparison of PECA-pS and PECA-R with the ODE-based model.

### GP curve fitting for smoothing and imputation of missing values

Before any data analysis module from PECAplus was applied, we used a smooth curve fitting procedure to mRNA and protein time series data. Assuming that the observed data points are realizations from a GP model, we optimized the parameters governing the Gaussian kernel and noise variance parameter empirically based on multiple data sets. After fitting a curve onto the time series data of each molecular type, we replace the observed intensity values with the predicted values from the GP model. If an intensity value is missing at a particular time point, the value is imputed by the posterior mean of the curve at that time point, which yields the most likely intensity value given other values in the neighboring time points according to the estimated GP model. The details of the mathematical model can be found in the Supplementary Information.

### Gene set analysis

We implemented the test for time-specific enrichment of biological functions in a gene list, which is selected by a user-provided threshold of CPS scores. At the threshold, we make a list of genes for which rate ratios or rate parameters scored above the CPS threshold at each time point, and perform hypergeometric tests for all relevant biological functions in three different ways: the ones for which the rate or rate ratio parameter increased (up-regulation), decreased (down-regulation), or changed in any direction (significant-regulation). The background gene list is automatically adjusted to the genes included in the entire data. The user can specify the range of functions to test enrichment for, such as the minimum number of significant genes in the function and the number of genes in the function (e.g., size of a GO term). The software package contains GO and CPDB annotations mapping to mouse and human genes.

### PECA-N model

PECA-N employs the same statistical model as the original PECA in Teo et al.^11^ with a notable exception. In PECA Core, the prior probability of change point in a rate ratio parameter at time *t* is the same for every gene, which is estimated from the data across all genes. In PECA-N, we employ the MRF prior,^19^ where the prior probability of change point in a gene is adjusted by the change point status of other first degree neighbor genes in a user-provided biological network. To identify the neighbor genes, we used the protein–protein interaction data from the STRING database.^20^ See Supplementary Information for the details of the model and estimation procedure.

### PECA-pS model

PECA-pS model uses pulsed-SILAC data for the proteomic data to estimate synthesis and degradation rates separately (up to a constant) and infer regulatory changes across the time points in synthesis and degradation separately. The model for the synthesis rate parameter takes the amount of mRNA available at the beginning of each time period into estimation, while the model for the degradation rate is formulated as a function of protein abundance at the beginning of each time period and the rate parameter, disregarding the abundance of mRNA. See Supplementary Information for the details of the model and estimation procedure.

### PECA-R model

PECA-R aims to estimate synthesis and degradation rates separately from proteomic expression data (along with mRNA). The model expresses the total concentration change as a sum of increase in concentration due to new synthesis and decreased due to degradation. The synthesis and degradation rate parameters are estimated under the following assumptions:

(i) When the total concentration increases, it is due to the increase in the synthesis rate as long as the mRNA concentration did not rise sufficiently high to explain the protein concentration at a fixed synthesis rate;
(ii) When the total protein concentration decreases, it is due to the increase in the degradation rate as long as the mRNA concentration did not drop sufficiently to explain the protein concentration change at a fixed degradation rate.

The reason for imposing those assumptions on the parameter space is straightforward. In label-free or TMT data, we only observe total protein changes, without separate abundance measurements for newly synthesized and existing proteins. Hence when the protein concentration changes, this model has to make a decision as to whether the synthesis rate and/or the degradation rate changed, considering the changes in mRNA concentration.

Since the total protein concentration changes can be explained by infinitely many combinations of the two rate parameters, the statistical significance score (CPS) is often more diluted in PECA-R than those values from PECA-pS. However, the PECA-pS model is not applicable unless pulse-labeled samples are available, and PECA-R is the next best option for non-pulse-labeled data within the PECAplus package if the estimation of synthesis and degradation is the ultimate aim of the analysis. See Supplementary Information for the details of the model, estimation procedure, and the restricted parameter space.

### Computation time and data availability

PECAplus can be downloaded from https://github.com/PECAplus (Apache 2.0 license), along with a tutorial and example data sets. The code requires a Windows, Mac OS X or Linux/Unix environment and enables the advanced access to the entire functionality of the tool. Second, the software package is available as a plugin to the widely used Perseus software (version 1.6.0.2), which was developed as a multi-functional platform for proteomics data analysis.^12^ This platform enables researchers without bioinformatics background to use PECAplus without any code manipulation. The Perseus platform also allows for easy visualization of the output. Run times of different modules vary by computer specifications and also depend on dataset size. With a ~3000 gene input dataset as discussed here used with default settings on an Windows 10 Home with Intel(R) Core(TM) i7-4710HQ CPU @2.50 GHz, 16 GB DDR3L SDRAM platform, the GP, PECA Core, PECA-pS, and PECA-R modules required ~1 h analysis time. The GSA module produces results instantaneously.

### Data availability

The ER stress data is from DatasetEV1 in the Supporting Information of Cheng et al.^4^ The LPS stimulation data is from Tables S1 and S2 in the Supporting Information of Jovanovic et al.^3^ The portion of the data used in this paper are provided as example data to illustrate software  reproducibility.

## Electronic supplementary material

Supplementary Information

## Electronic supplementary material

**Supplementary information** accompanies the paper on the *npj Systems Biology and Applications* website (10.1038/s41540-017-0040-1).

## References

[1] Schwanhausser B (2011). Global quantification of mammalian gene expression control. Nature.

[2] Lee MV (2011). A dynamic model of proteome changes reveals new roles for transcript alteration in yeast. Mol. Syst. Biol..

[3] Jovanovic M (2015). Immunogenetics. Dynamic profiling of the protein life cycle in response to pathogens. Science.

[4] Cheng Z (2016). Differential dynamics of the mammalian mRNA and protein expression response to misfolding stress. Mol. Syst. Biol..

[5] Liu TY (2017). Time-resolved proteomics extends ribosome profiling-based measurements of protein synthesis dynamics. Cell Syst..

[6] Lahtvee PJ (2017). Absolute quantification of protein and mRNA abundances demonstrate variability in gene-specific translation efficiency in yeast. Cell Syst..

[7] Schwanhausser B, Gossen M, Dittmar G, Selbach M (2009). Global analysis of cellular protein translation by pulsed SILAC. Proteomics.

[8] Ong SE (2002). Stable isotope labeling by amino acids in cell culture, SILAC, as a simple and accurate approach to expression proteomics. Mol. Cell. Proteom..

[9] Ross PL (2004). Multiplexed protein quantitation in Saccharomyces cerevisiae using amine-reactive isobaric tagging reagents. Mol. Cell. Proteom..

[10] Thompson A (2003). Tandem mass tags: a novel quantification strategy for comparative analysis of complex protein mixtures by MS/MS. Anal. Chem..

[11] Teo G, Vogel C, Ghosh D, Kim S, Choi H (2014). PECA: a novel statistical tool for deconvoluting time-dependent gene expression regulation. J. Proteome Res.

[12] Tyanova S (2016). The Perseus computational platform for comprehensive analysis of (prote)omics data. Nat. Methods.

[13] DeJesus, R. et al. Functional CRISPR screening identifies the ufmylation pathway as a regulator of SQSTM1/p62. *Elife* **5**, pii: e17290 (2016). 10.7554/eLife.17290 PMC4924995 27351204

[14] Homma K (2013). SOD1 as a molecular switch for initiating the homeostatic ER stress response under zinc deficiency. Mol. Cell.

[15] Troyanskaya O (2001). Missing value estimation methods for DNA microarrays. Bioinformatics.

[16] Ashburner M (2000). Gene ontology: tool for the unification of biology. The Gene Ontology Consortium. Nat. Genet..

[17] Kamburov A, Wierling C, Lehrach H, Herwig R (2009). ConsensusPathDB—a database for integrating human functional interaction networks. Nucleic Acids Res..

[18] Wei Z, Li H (2007). A Markov random field model for network-based analysis of genomic data. Bioinformatics.

[19] J B (1986). On the statistical analysis of dirty pictures. J. R. Stat. Soc. B.

[20] Jensen LJ (2009). STRING 8—a global view on proteins and their functional interactions in 630 organisms. Nucleic Acids Res..

