# Supplementary material for: PECAplus: statistical analysis of time-dependent regulatory changes in dynamic single-omics and dual-omics experiments
Source: NPJ Syst Biol Appl. 2017 Dec 19;4:3. doi: 10.1038/s41540-017-0040-1 (PMC5736550; doi:10.1038/s41540-017-0040-1)
Supplement: Supplementary file 1 — Supplementary Information [file 41540_2017_40_MOESM1_ESM.pdf]

# Supplementary Information for "PECAplus: statistical analysis of time-dependent regulatory changes in dynamic single- and dual-omics experiments"

Guoshou Teo
Center for Genomics and Systems Biology, Department of Biology
New York University, New York, NY, USA

Yunbin Zhang
College of Arts and Science
New York University, New York, NY, USA

Christine Vogel*
Center for Genomics and Systems Biology, Department of Biology
New York University, New York, NY, USA

Hyungwon Choi*
Institute of Molecular and Cell Biology, ASTAR
Saw Swee Hock School of Public Health, National University of Singapore, Singapore

*To whom all correspondence should be addressed. Email: hwchoi@nus.edu.sg or cvogel@nyu.edu

# Contents

**1 Supplementary Figures** **3**

**2 Gaussian Process model for smoothing and imputation** **8**
2.1 Model description . . . . . . . . . . . . . . . . . . . . . . . . . . . . . . . . . 8
2.2 Gaussian kernel and tuning parameters . . . . . . . . . . . . . . . . . . . . . . 8
2.3 Smoothing and missing data imputation . . . . . . . . . . . . . . . . . . . . . 9

**3 PECA-N: Incorporating biological network information into the PECA model** **10**
3.1 Likelihood and prior distributions . . . . . . . . . . . . . . . . . . . . . . . . . 10
3.2 Definition of module information . . . . . . . . . . . . . . . . . . . . . . . . . 11
3.3 MCMC sampler . . . . . . . . . . . . . . . . . . . . . . . . . . . . . . . . . . . 11

**4 PECA-pS: A PECA model when pulse-labelled proteomic data is available** **13**
4.1 Likelihood and prior distributions . . . . . . . . . . . . . . . . . . . . . . . . . 14
4.2 MCMC sampler . . . . . . . . . . . . . . . . . . . . . . . . . . . . . . . . . . . 14

**5 PECA-R: Inferring synthesis and degradation rates from proteomic data not derived from a pulse-labeling experiment** **16**
5.1 Likelihood and prior distributions . . . . . . . . . . . . . . . . . . . . . . . . . 17
5.2 MCMC sampler . . . . . . . . . . . . . . . . . . . . . . . . . . . . . . . . . . . 18

# 1 Supplementary Figures

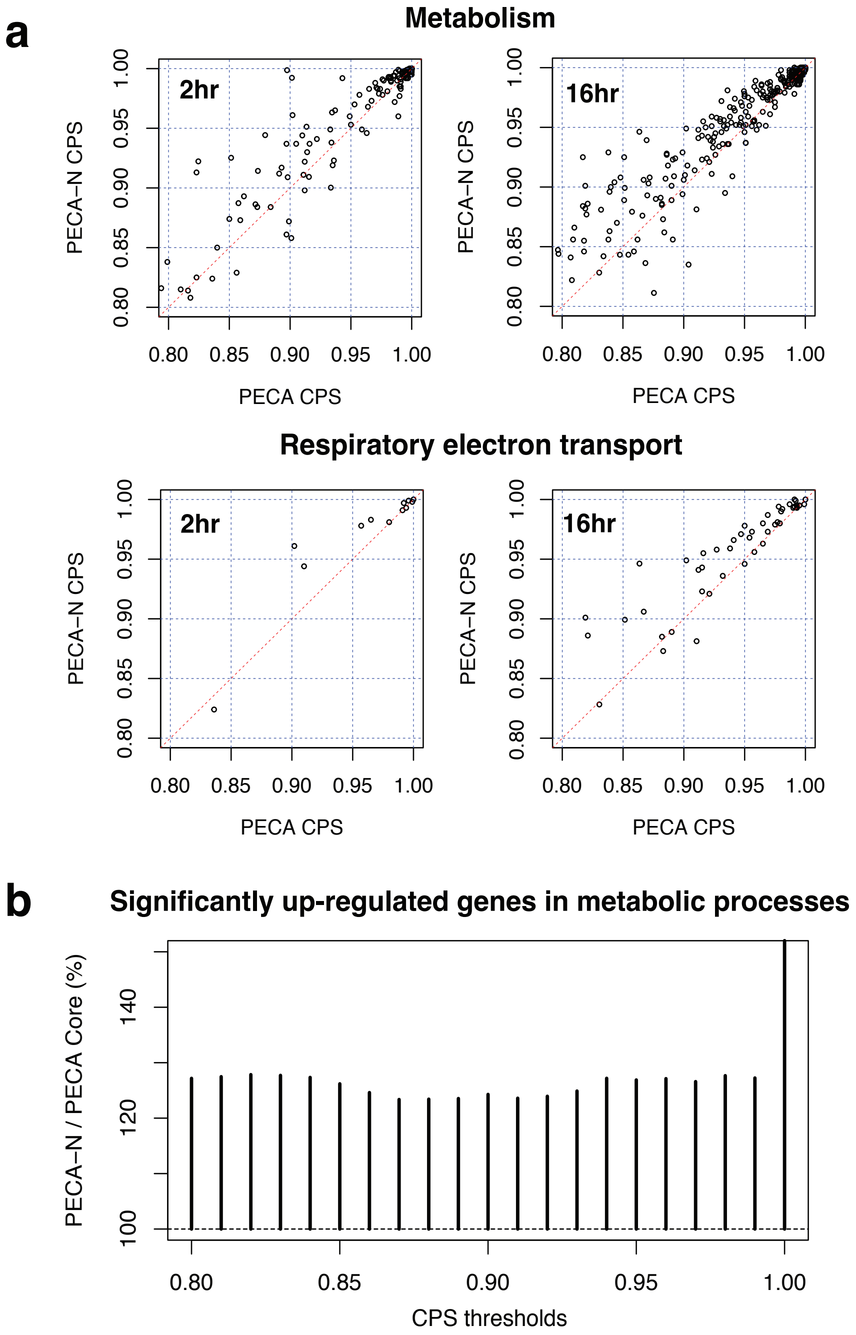


**Supplementary Figure 1**. **a**. PECA-N produced greater CPS scores than PECA Core for the genes belonging to metabolism and respiratory electron transport at 16 hrs at the RNA level. **b**. The ratio of the number of significantly up-regulated RNAs between PECA-N and PECA core at the same CPS score thresholds among 1720 genes in the metabolic processes.

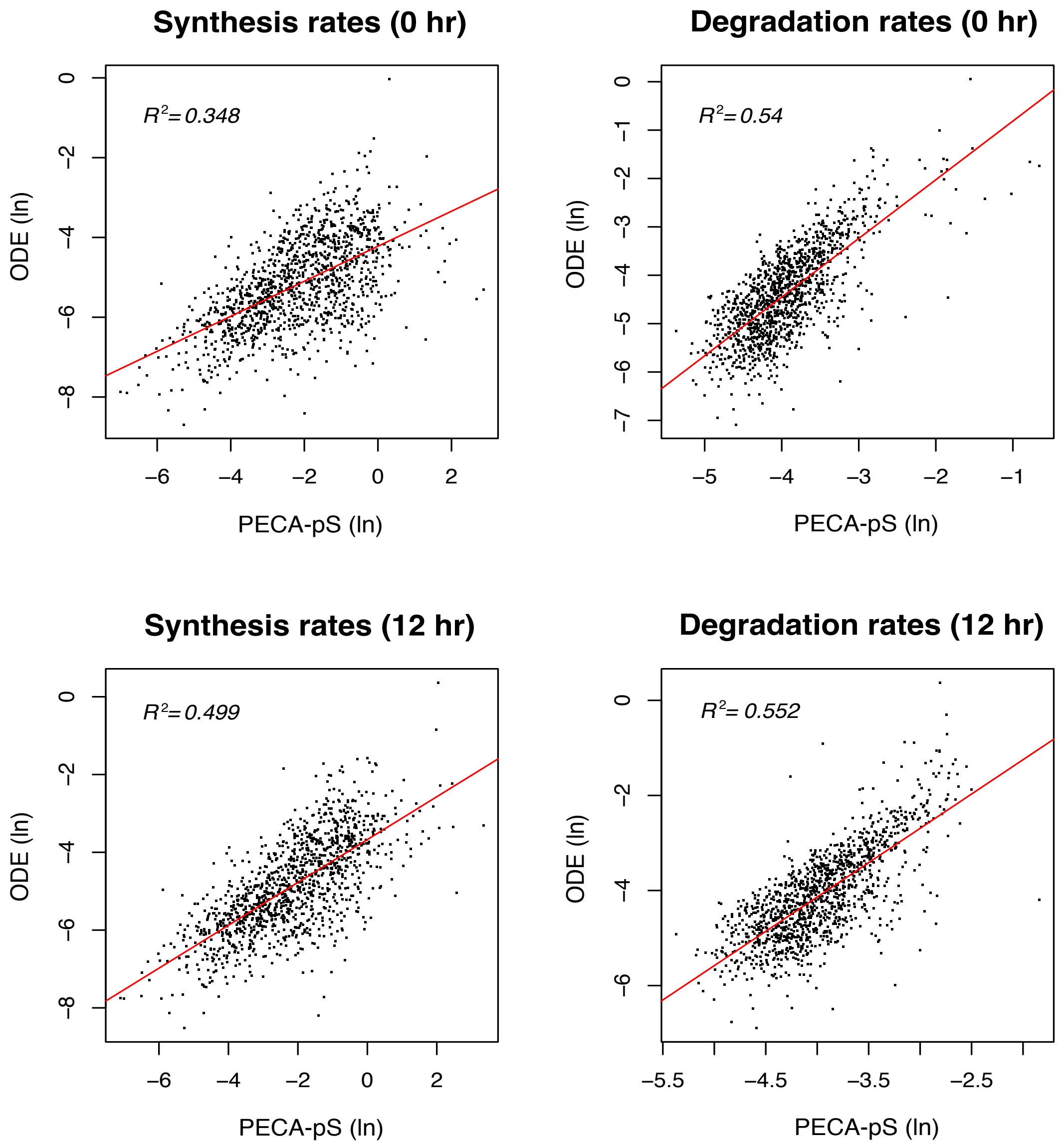


**Supplementary Figure 2**. Comparison of rate parameter estimates between the ODE-based approach (Jovanovic *et al.*) and PECA-pS in the synthetic LPS response data set. For PECA-pS (horizontal axis), the rate parameters in the adjacent time intervals at 0 and 12 hours were used for comparison with the rates from the ODE-based approach at the respective time points.

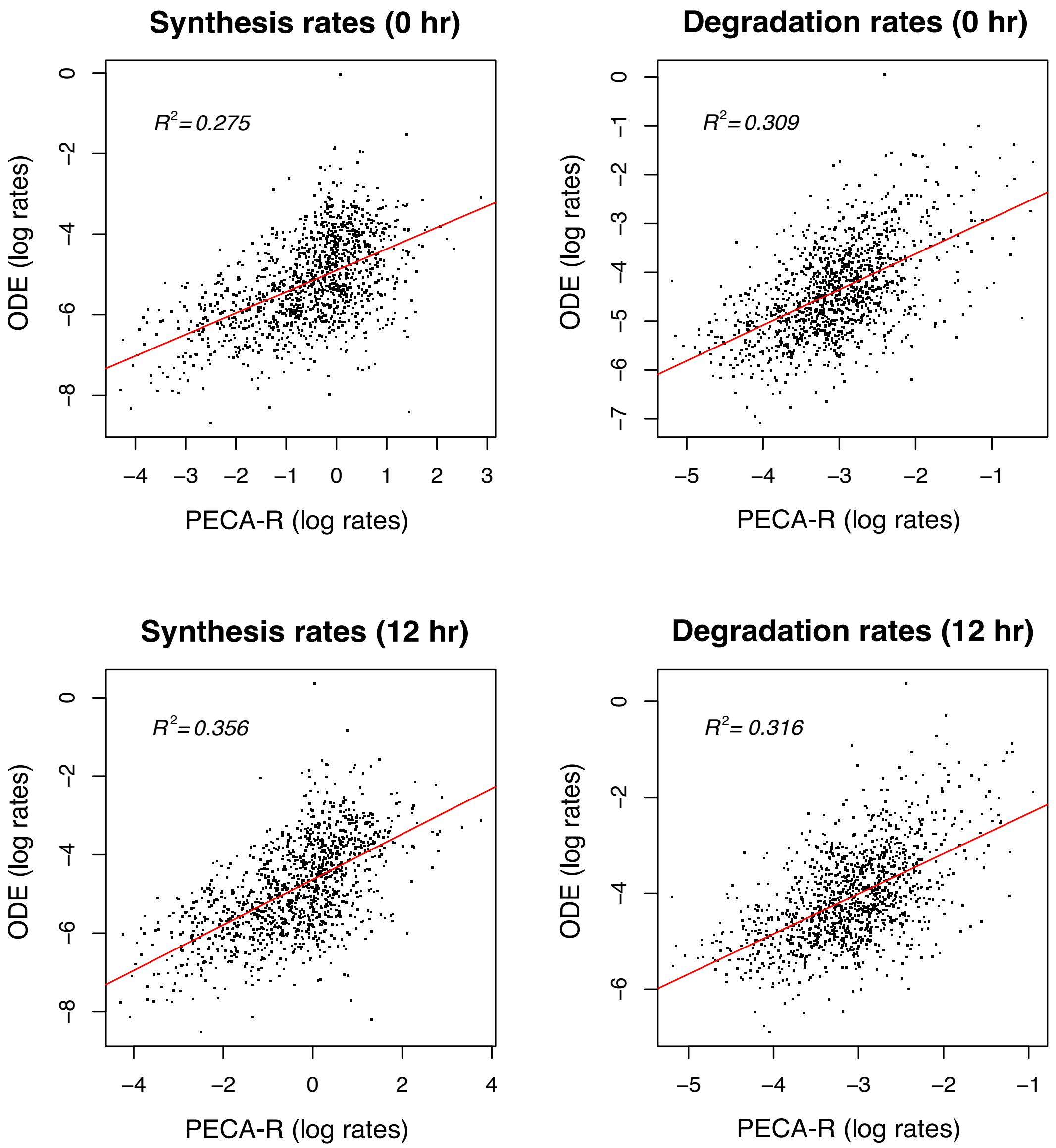


**Supplementary Figure 3**. Comparison of rate parameter estimates between the ODE-based approach (Jovanovic *et al.*) and PECA-R in the synthetic data set. For PECA-R (horizontal axis), the rate parameters in the adjacent time intervals at 0 and 12 hours were used for comparison with the rates from the ODE-based approach at the respective time points.

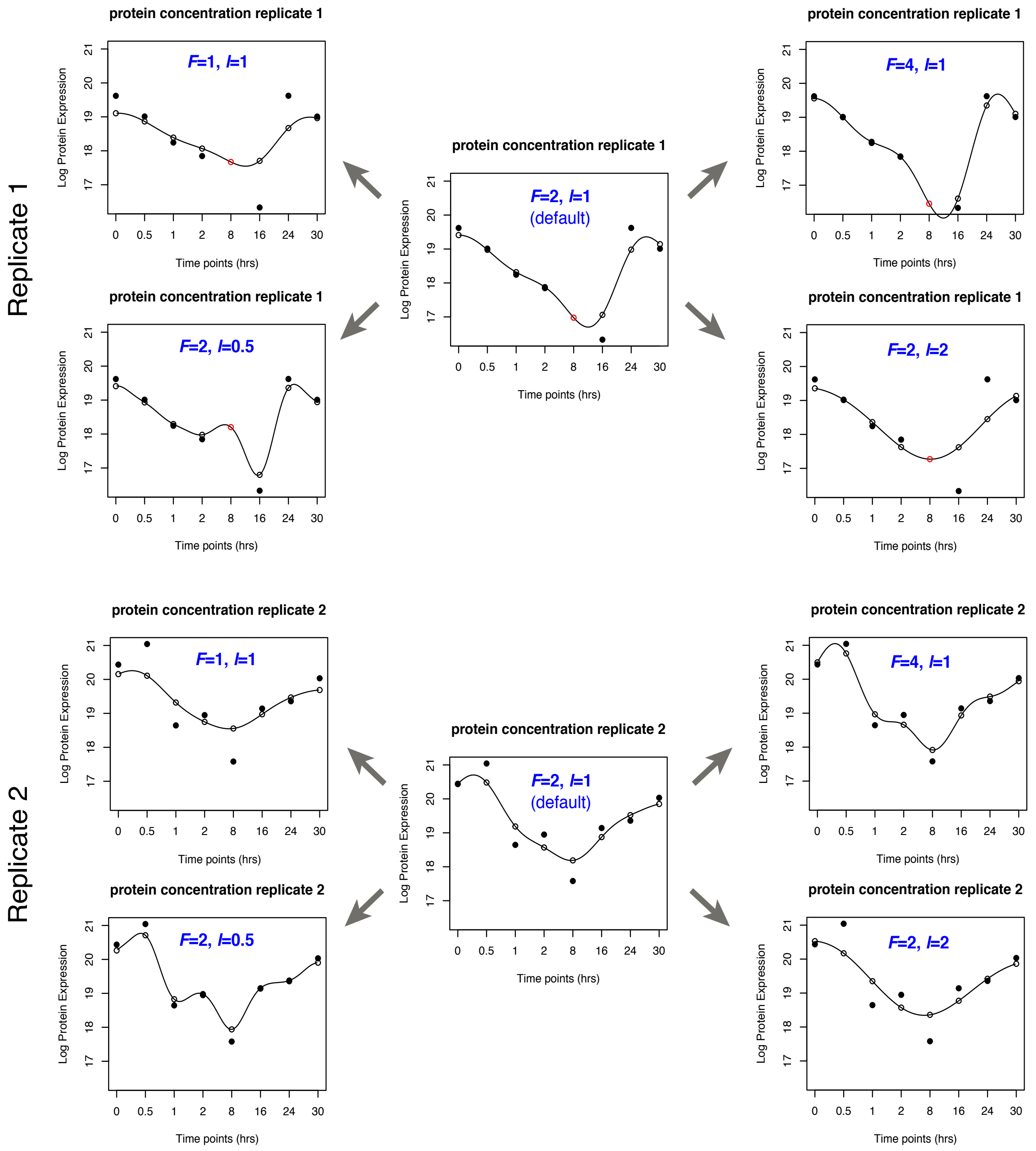


**Supplementary Figure 4**. GP smoothing is robust to variation of parameters $F$ and $\ell$ within the proposed range. We illustrate the impact of the parameters for the SLC39A14 gene (two biological replicates). The two parameters work in concert to control the overall smoothness of curves. We optimized parameters for small to medium sized time series that are typical for biological experiments. We varied each parameter from small to large with respect to the default value while fixing the other parameter at default value. The covariance weight parameter $F$ was varied from 1 to 4 while fixing $\ell = 1$. Local variance parameter $\ell$ was varied from 0.5 to 2 while fixing $F = 2$.

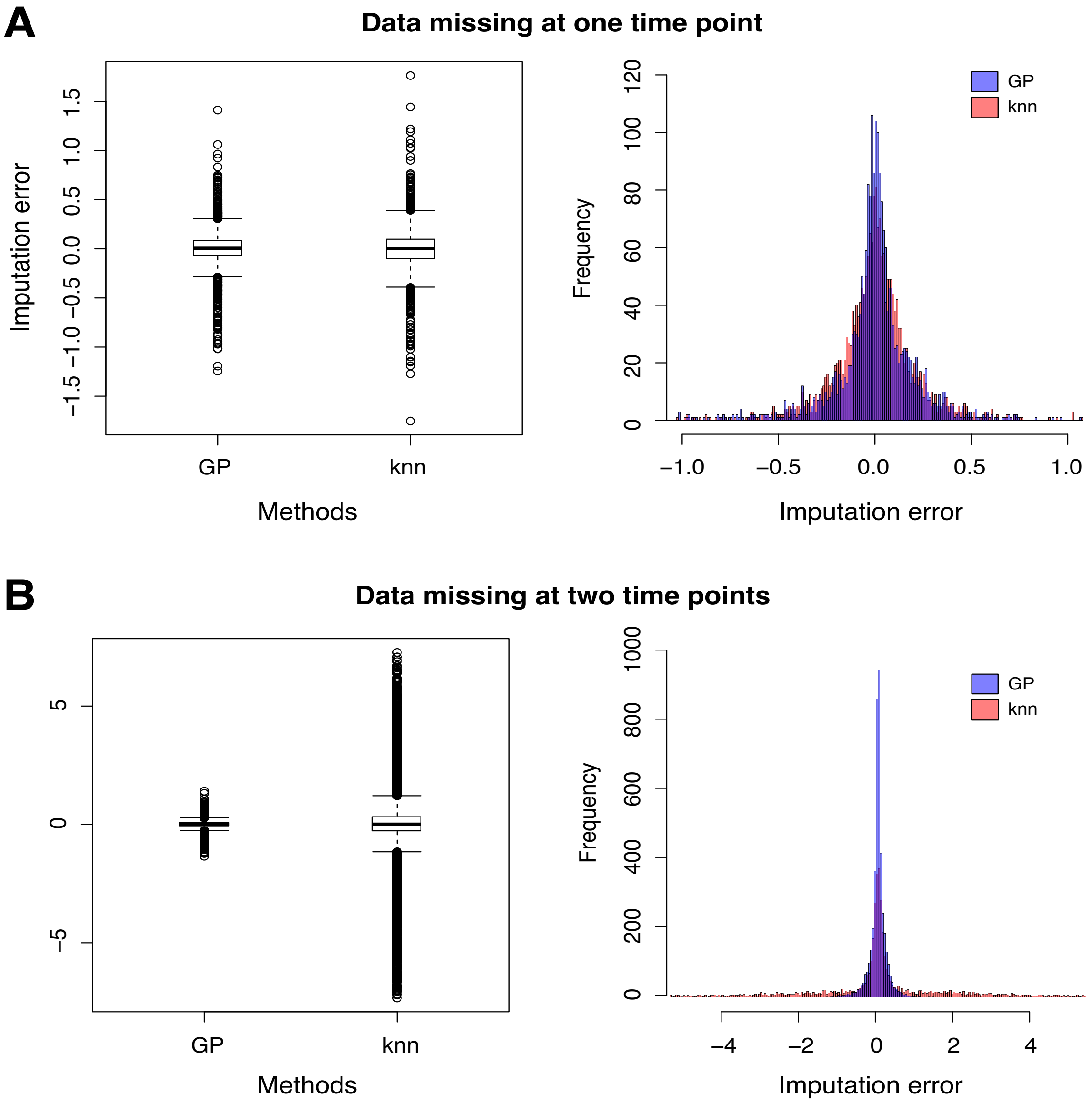


**Supplementary Figure 5**. The GP-based imputation (used in PECAplus) is more robust to leave-one-out and leave-two-out tests than K-nearest neighbor (knn) imputation. In the ER stress data, we removed one or two intensity values from randomly selected time points for each gene individually, and challenged both methods to impute the values. We then computed imputation errors as the difference between imputed values and observed (but erased) values. The panels show the distributions of the imputation errors across all genes at all time points. The distributions are centered more narrowly around 0 (no error) for GP smoothing than for knn imputation.

# 2 Gaussian Process model for smoothing and imputation

## 2.1 Model description

In this section, we provide detailed description of the Gaussian Process (GP) model [5], which smoothes rugged time series data and imputes missing observations in PECAplus. A time series for a gene, whether it is mRNA or protein, is expressed as a function of time $f(h)$ with the following kernel:

$$f(h) \sim GP(0, k(h, h'))$$
$$k(h, h') = E[(f(h))(f(h'))^T].$$

For any finite set of time points, we assume that the distribution of the function $f$ between any two time points $h_i$ and $h_j$ follows Gaussian distribution

$$p(\mathbf{f}|\mathbf{h}) = \mathcal{N}(\mathbf{f}|0, \mathbf{K}),$$

that is $K_{ij} = k(h_i, h_j)$ denotes the Gaussian kernel specified below. Thanks to this flexible definition of the stochastic process, GP is a widely used, highly flexible technique for inference of time series data in many areas of application.

## 2.2 Gaussian kernel and tuning parameters

The Gaussian kernel is defined as

$$\begin{aligned} k_y(h_p, h_q) =& F^2 \cdot \sigma_y^2 \cdot \exp\left\{-\frac{1}{2\ell^2}(h_p - h_q)^2\right\} + \sigma_y^2 \cdot I(p = q) \\ \boldsymbol{\theta} =& (F^2, \ell^2, \sigma_y^2) \end{aligned}$$

The value of $\sigma_y^2$ does not affect the value of $\bar{f}_*$ so we simplify the kernel to

$$\begin{aligned} k_y(h_p, h_q) =& F^2 \cdot \exp\left\{-\frac{1}{2\ell^2}(h_p - h_q)^2\right\} + I(p = q) \\ \boldsymbol{\theta} =& (F^2, \ell^2) \end{aligned}$$

$F$ and $\ell$ values are user-defined parameters. These two parameters jointly control the shape of smoothed curve, with subtle difference in their roles: the parameter $F$ controls the amount of correlation between time points (not necessarily observation time points), whereas the parameter $\ell$ controls the variability of values in neighboring time points, i.e. ruggedness of curve in local temporal neighborhoods. For this reason, we call $F$ the absolute covariance weight parameter and $\ell$ the local variance parameter.

If the user sets a small value of $F$ (e.g. $0.1 \sim 0.5$), then the model assumes that the expression values in any two time points are less correlated. This leads the model to think that the variation along the time course is more attributable to random noise than systematic changes, and therefore results in a flat curve. By contrast, a large $F$ (e.g. $2 \sim 5$) will honor correlated changes along time and produce a curve closer to the original observations with reduced amount of smoothing.

Meanwhile, the parameter $\ell$ controls the amount of fluctuations allowed in neighboring time points. If the user sets a small value of $\ell$ (e.g. $0.1 \sim 0.5$), then the curve has to be fit tightly to the observed data, therefore losing flexibility in the curve. This tends to produce more locally rugged patterns (less smoothed). By contrast, if the user sets a large value of $\ell$ (e.g. $2 \sim 5$),

then the curve no longer has to tightly fit the observed values and can have smoother shape along the time course.

In the current implementation, we set the default values at $(F, \ell) = (2, 1)$. Supplementary Figure 4A shows the impact of the covariance weight parameter $F$ on the smoothed curve when the local variance parameter $\ell$ is fixed at 1. As explained above, at a fixed value of $\ell$, larger values of $F$ render the curve to be more "correlated" in nearby time points (nearby observed values), i.e. closely fitting the observed data points. Supplementary Figure 4B shows that smaller $\ell$ produces less smooth a curve closely fitting the observed data. We have chosen $F = (2, 1)$ based on empirical evidence over a number of data sets we analyzed, and we recommend that these values are not adjusted too much by users who are not familiar with the modeling. However, the python script automatically produces these plots, which helps the user make a more informed decision on optimal parameters for their own data.

## 2.3 Smoothing and missing data imputation

The above model can be used to calculate the expected (de-noised) expression level $E(y)$ at any time point $x$ for both smoothing and imputation. We first use all observed data points $\{(h_i, y_i), i = 1 : N\}$ to train the model

$$\begin{aligned} y =& f(h) + \epsilon, \quad \epsilon \sim \mathcal{N}(0, \sigma_y^2) \\ k_y(h_p, h_q) =& \text{cov}[y_p, y_q | h_p, h_q] = k(h_p, h_q) + \sigma_y^2 I(p = q) \\ \mathbf{K}_y =& \text{cov}[\mathbf{y}|\mathbf{h}] = \mathbf{K} + \sigma_y^2 \mathbf{I}_N \end{aligned}$$

where $\{h_i\}$ are the observation time points and $\{y_i\}$ are the observed expression value of a molecule. If expression data is missing at time $h_*$, then we impute with posterior mean $\bar{f}_*$ to the time point:

$$\begin{aligned} \bar{f}_* =& \mathbf{k}_*^T \mathbf{K}_y^{-1} \mathbf{y} \\ \mathbf{k}_* =& [k(h_*, h_1), \ldots, k(h_*, h_N)]. \end{aligned}$$

We apply the same to each observation time point with non-missing data to perform smoothing on the entire time series.

To evaluate whether the imputation scheme is efficient, we performed a numerical study comparing GP-based imputation with K-nearest neighbor (KNN)-based imputation. To do this, we took the mRNA data from the ER stress study and modified the data as follows. We randomly selected one time point in each gene and erased the observed intensity value, making it a missing data (Supplementary Figure 5A). After erasing one data point per gene, we challenged both methods to impute the values. To evaluate the performance, we compared the difference between imputed values and true underlying values. Supplementary Figure 5A shows that GP-based smoothing is overall superior to the KNN method, giving smaller imputation errors. This performance difference became more pronounced when we erased two data points per gene (Supplementary Figure 5B).

# 3 PECA-N: Incorporating biological network information into the PECA model

PECA-N employs the same statistical model as the original PECA Core [6]. In PECA, the prior probability of change point in a rate ratio parameter at time t is the same for every gene, which is estimated from the data across all genes. In PECA-N, we employ the Markov random field (MRF) prior [2, 7], where the prior probability of change point in a gene is adjusted by the change point status of other first degree neighbor genes in a user-provided biological network. We used the protein-protein interaction data from the STRING database [4]. To estimate the model parameters, we constructed a Markov chain Monte Carlo (MCMC) sampler that combines standard Metropolis-Hastings updates and dimension switching updates in the form of reversible-jump MCMC [3]. The following section describes the full details on the PECA-N model and Bayesian inference of the model parameters.

## 3.1 Likelihood and prior distributions

First, suppose that the experiment has measurements for $I$ genes across $T$ times points in $N$ biological replicates. Then, the likelihood of the entire PECA Core model is

$$\text{(likelihood)} = \prod_{i=1}^{I}\prod_{j=1}^{N}\prod_{t=0}^{T} \frac{1}{y_{jit}\tau_i\sqrt{2\pi}} \exp\left[-\frac{1}{2\tau_i^2}(\ln(y_{jit}) - \ln(\eta_{jit}))^2\right]$$

where

$$\eta_{jit} = \eta_{ji0} + \sum_{\ell=0}^{t-1} \Delta h_\ell \left(x_{ji\ell}\kappa'_{i\ell} - \eta_{ji\ell}(1-\kappa'_{i\ell})\right),$$

and $i$, $j$, and $t$ index gene, replicate, and time, respectively. We specify prior distributions that are the least subjective with wide variance parameters:

$$\begin{aligned} \eta_{ji0} &\sim \mathcal{N}(0, 100^2) \quad \text{for } j = 0, \ldots, N \\ \kappa'_{i\ell} &\sim \mathcal{U}(0,1) \quad \text{for } \ell = 0, \ldots, |\boldsymbol{C}_i| \\ \tau_i^{-2} &\sim \mathcal{G}(a_\tau, b_\tau) \end{aligned}$$

for fixed $\boldsymbol{C}_i$ for all $i$, where $\mathcal{N}$, $\mathcal{U}$, $\mathcal{G}$ denote normal, uniform, and gamma distributions respectively. Here $\boldsymbol{C}_i$ is the change point set for gene $i$, i.e. the collection of time points in which rate ratios in adjacent time periods are different. This set is empty when the rate ratio remains constant over time, and becomes a non-empty set when there is at least one change point. Accordingly, $|\boldsymbol{C}_i|$ denotes the number of change points in gene $i$, which is a random variable on its own and will be inferred during the estimation procedure. We impose the following prior for $\boldsymbol{C}_i$:

$$\pi(\boldsymbol{C}_i) \propto \varphi_{it}^{|\boldsymbol{C}_i|}(1-\varphi_{it})^{T-1-|\boldsymbol{C}_i|},$$

where $\varphi_{it}$ is the probability that gene $i$ has a change point probability at time $t$, i.e. the rate ratios $\kappa'_{i,t-1}$ and $\kappa'_{it}$ are different. The posterior expectation of this parameter becomes the final **change point score (CPS)** of gene $i$ at time $t$, i.e. $P(\kappa'_{i,t-1} \neq \kappa'_{it}|\mathbf{x}, \mathbf{y})$, where $\mathbf{x}$ and $\mathbf{y}$ denote the two layers of expression data.

The difference between PECA Core and PECA-N is how $\varphi_{it}$ is defined and estimated. In PECA Core, the parameter is estimated solely based on the data for gene $i$. In PECA-N, by contrast, this parameter is estimated with the help of the network information (or module information) from related genes is incorporated (see below), using the MRF prior.

## 3.2 Definition of module information

There are different types of network information in the literature. Most biological networks are provided as a list of gene pairs that interact with one another. One can convert gene groups (e.g., Gene Ontology [1] or GO terms) into this format by forming cliques for a group of genes, i.e. enumerating all pair-wise interactions in the group. We refer both formats of network data as *module information* hereafter.

## 3.3 MCMC sampler

When the module information is utilized, PECA-N will run the MCMC sampler twice. The first MCMC sampling will be done following the sampler of PECA Core. On the completion of the first MCMC run, we estimate the MRF prior coefficient by finding a maximizer of

$$\prod_i \frac{exp(\mathcal{I}(CPS_{it} > .5) \cdot F_{it})}{1 + exp(F_{it})}$$

with respect to $\gamma_t$, where

$$F_{it} = \gamma_{t,0} + \gamma_{t,1} \frac{\sum_{j \in \partial i} \mathcal{I}(CPS_{jt} > .5)}{\#\text{neighbors of gene i}}$$

for all $t$. On the second MCMC sampling set

$$logit(\varphi_{it}) = F_{it}$$

for all $i$ and $t$.

In summary, the prior can be written as

$$(\text{prior}) \;\propto\; \prod_{i=1}^{I} \left\{ \frac{b_\tau^{a_\tau}}{\Gamma(a_\tau)} (\tau_i^2)^{-a_\tau - 1} e^{-\frac{b_\tau}{\tau_i^2}} \cdot \prod_j \phi(\tfrac{\eta_{ji0}}{100}) \cdot \varphi^{|\boldsymbol{C}_i|} (1-\varphi)^{T-1-|\boldsymbol{C}_i|} \right\}$$

where the prior for $\{\kappa'_{it}\}$ is omitted conditional on the fact that the parameters are uniformly distributed on unit interval [0,1], and $\phi$ denotes standard normal density.

The model parameters are updated in the following order:

$$\{\eta_{ji0}\}_{j=0}^{N} \to \tau_i^2 \to \{\kappa'_{it}\}_{t=0}^{T-1} \to \boldsymbol{C}_i$$

for all $i$. This whole cycle is repeated for 1,000 iterations for burn-in period and $M = 10,000$ iterations for the main iteration with thinning of 10 samples, in both simulation and data analysis sections that follow. We use hat and tilde symbols to denote current and proposed values respectively.

1. We first start with $\eta_{ji0}$. We run the random walk Metropolis algorithm chain in the log-space by generating the proposal $log(\tilde{\eta}_{ji0})$ from $\mathcal{N}(log(\hat{\eta}_{ji0}), 1)$ or equivalently $\tilde{\eta}_{ji0}$ from $\hat{\eta}_{ji0} \exp(\mathcal{N}(0,1))$. Running the chain in the log-space alleviates the need to tune for step sizes for each $\eta_{ji0}$. Since this parameter is involved in the expected values of $y_{ijt}$ at all time points, the likelihood has to be evaluated at all time points for updating each of these parameters.

2. Next, we draw the variance parameter $\tau_i^2$ by Gibbs sampling from inverse gamma distribution $\mathcal{IG}(a_\tau + N(T+1)/2, b_\tau + \sum_{j,t}(y_{jit} - \eta_{jit})^2/2)$.

3. Next, we draw $\{\kappa^s_{i\ell}\}$ for $\ell = 0, \ldots, |\boldsymbol{C}_i^{(s)}|$ under the fixed $\boldsymbol{C}_i^{(s)}$ for each protein $i$. We run the chain in the logit-space, i.e. draw a proposal value $logit(\tilde{\kappa}^s_{i\ell})$ from $\mathcal{N}(logit(\hat{\kappa}^s_{i\ell}), 1)$ and accept or reject afterwards.

4. Finally, we update the change point set $\boldsymbol{C}_i$. There are two different moves: birth of a new change point and removal (death) of an existing change point. Since these two moves are reversible in notation, we just describe the birth move here. Suppose that $\hat{\kappa}'_{i\ell}$ covers a time period $(h_t, h_{t+m})$ that contains at least one observation time(s). Then we propose a birth of a new change point $h^* \in \{h_{t+1}, \ldots, h_{t+m-1}\}$ within the interval (chosen from one of the intermediate time points) and break the current rate parameter into two daughter parameters, namely $(\tilde{\kappa}'_{i\ell}, \tilde{\kappa}'_{i,\ell+1})$ where it is required to meet

$$(h^* - h_t) \cdot \text{logit}(\tilde{\kappa}'_{i\ell}) + (h_{t+m} - h^*) \cdot \text{logit}(\tilde{\kappa}'_{i,\ell+1}) = (h_{t+m} - h_t) \cdot \text{logit}(\hat{\kappa}'_{i\ell})$$

with a random perturbation such that

$$\frac{\tilde{\kappa}'_{i,\ell+1}}{1 - \tilde{\kappa}'_{i,\ell+1}} = \frac{1-u}{u} \frac{\tilde{\kappa}'_{i\ell}}{1 - \tilde{\kappa}'_{i\ell}},$$

with $u \sim \text{Uniform}(0, 1)$. Under this transformation, the Jacobian is $\frac{(\tilde{\kappa}'_{i\ell}(1-\tilde{\kappa}'_{i\ell}) + \tilde{\kappa}'_{i,\ell+1}(1-\tilde{\kappa}'_{i,\ell+1}))^2}{\hat{\kappa}'_{i\ell}(1-\hat{\kappa}'_{i\ell})}$ for $(\hat{\kappa}'_{i\ell}, u) \to (\tilde{\kappa}'_{i\ell}, \tilde{\kappa}'_{i,\ell+1})$. Hence the Metropolis-Hastings ratio for the birth move just equals the posterior ratio times the Jacobian since the acceptance probability of this proposal is

$$\min\{1, \text{likelihood ratio} \times \text{prior ratio} \times \text{proposal ratio} \times \text{Jacobian}\},$$

where the prior and proposal ratios are the ratios of Uniform distribution over unit intervals. Then the Metropolis-Hastings ratio becomes

$$\prod_{j,t} \left[ \exp\left\{ -\frac{1}{2\tau_i^2} (\ln(y_{jit}) - \ln(\eta_{jit}))^2 \right\} \right] \frac{\varphi}{1-\varphi} \frac{(\tilde{\kappa}'_{i\ell}(1 - \tilde{\kappa}'_{i\ell}) + \tilde{\kappa}'_{i,\ell+1}(1 - \tilde{\kappa}'_{i,\ell+1}))^2}{\hat{\kappa}'_{i\ell}(1 - \hat{\kappa}'_{i\ell})}.$$

# 4 PECA-pS: A PECA model when pulse-labelled proteomic data is available

PECA-pS uses pulsed-SILAC data for the proteomic data to estimate synthesis and degradation rates and infer regulatory changes across the time points in synthesis and degradation separately. The model for the synthesis rate parameter takes the amount of mRNA available at the beginning of each time period into estimation, while the model for the degradation rate is formulated as a function of protein abundance at the beginning of each time period and the rate parameter, disregarding the abundance of mRNA.

Suppose that we have parallel mRNA and protein expression data (with medium (M) channel signal and heavy (H) channel signal) $\boldsymbol{X} = \{x_{jit}\}$, $\boldsymbol{Y}^{(M)} = \{y_{jit}^{(M)}\}$ and $\boldsymbol{Y}^{(H)} = \{y_{jit}^{(H)}\}$ for protein $i = 1, \ldots, I$ in replicates $j = 1, \ldots, N$ observed over time points $(h_0, \ldots, h_T)$. Time $h_0$ indicates the time point at which or before the samples are treated or the baseline of subsequent time points. We assume that the protein expression measurements follow log normal distributions

$$
\begin{aligned}
y_{jit}^{(M)} &\sim \mathcal{LN}\left(\ln(\eta_{jit}^{(M)}), (\tau_i^{(M)})^2\right) \\
y_{jit}^{(H)} &\sim \mathcal{LN}\left(\ln(\eta_{jit}^{(H)}), (\tau_i^{(H)})^2\right)
\end{aligned}
$$

after proper normalization of the data. Our goal is to infer the protein synthesis rate $\kappa_{it}^s$ and the degradation rate $\kappa_{it}^d$ during the interval $(h_t, h_{t+1})$ of length $\Delta h_t = (h_{t+1} - h_t)$ for protein $i$. More importantly, the mean parameters are related between adjacent time points as follows:

$$
\eta_{ji,t+1}^{(M)} = \eta_{jit}^{(M)} + \Delta h_t \left(-\eta_{jit}^{(M)} \kappa_{it}^d\right) \eta_{ji,t+1}^{(H)} = \eta_{jit}^{(H)} + \Delta h_t f(x_{jit}) \kappa_{it}^s \tag{1}
$$

where

$$
f(x) = \log(1 + x)
$$

for $t = 0, 1, \ldots, T - 1$. This mathematical assumption was put in place after we noticed that, without such transformation, the majority of signals were detected mostly on highly abundant genes only. The transformation log(x+1) "de-sensitizes" the change point scores in very highly abundant genes only, especially when the data are very noisy.

To detect the change in these rate parameters, we formulated a change point model similar to PECA Core to describe the probability distribution of $\boldsymbol{\kappa}_i^s = (\kappa_{i0}^s, \cdots, \kappa_{i,T-1}^s)$ as follows. We first note that the synthesis rate $\kappa_{it}^s$ and the degradation rate $\kappa_{it}^d$ are always positive since they are rate parameters by definition. Second, unlike the PECA Core and PECA-N models, we define the change point set $\boldsymbol{C}_i$ for synthesis and degradation separately, since synthesis and degradation rates may not share the same change point in the same gene across time points. For gene $i$, let $\boldsymbol{C}_i^{(s)}$ and $|\boldsymbol{C}_i^{(s)}|$ denote the set of time points $\{t : \kappa_{i,t-1}^s \neq \kappa_{it}^s | 0, 1, \cdots, T - 1\}$ and the size of the set, respectively. If the synthesis rates of $\boldsymbol{\kappa}_i^s$ remained constant over time, $\boldsymbol{C}_i^{(s)}$ is an empty set; if synthesis rate values of $\boldsymbol{\kappa}_i^s$ in some time periods were different from others, $\boldsymbol{C}_i^{(s)}$ is the set of all intermediate time points from 1 to $T - 1$ with different adjacent rates. The change point set $\boldsymbol{C}_i^{(d)}$ for the degradation rate parameters $\boldsymbol{\kappa}_i^d$ are defined similarly. From this model, the CPS scores are computed for synthesis and degradation separately, by computing $P(\kappa_{i,t-1}^s \neq \kappa_{it}^s | \mathbf{x}, \mathbf{y}^{(H)})$ for the synthesis parameter and $P(\kappa_{i,t-1}^d \neq \kappa_{it}^d | \mathbf{x}, \mathbf{y}^{(M)})$ for the degradation parameter of each gene $i$ at time $t$.

### 4.1 Likelihood and prior distributions

First, the likelihood of the entire model can be written as

$$\begin{aligned}(\text{likelihood}) =& \prod_{i=1}^{I}\prod_{j=1}^{N}\prod_{t=0}^{T}\frac{1}{y_{jit}^{(M)}\tau_i^{(M)}\sqrt{2\pi}}\exp\left[-\frac{1}{2(\tau_i^{(M)})^2}(\ln(y_{jit}^{(M)})-\ln(\eta_{jit}^{(M)}))^2\right]\\ &\times\prod_{i=1}^{I}\prod_{j=1}^{N}\prod_{t=0}^{T}\frac{1}{y_{jit}^{(H)}\tau_i^{(H)}\sqrt{2\pi}}\exp\left[-\frac{1}{2(\tau_i^{(H)})^2}(\ln(y_{jit}^{(H)})-\ln(\eta_{jit}^{(H)}))^2\right]\end{aligned}$$

where

$$\begin{aligned}\eta_{jit}^{(M)} =&\eta_{ji0}^{(M)}+\sum_{\ell=0}^{t-1}\Delta h_\ell\left(-\eta_{ji\ell}^{(M)}\kappa_{i\ell}^d\right)\\ \eta_{jit}^{(H)} =&\eta_{ji0}^{(H)}+\sum_{\ell=0}^{t-1}\Delta h_\ell\left(f(x_{ji\ell})\kappa_{i\ell}^s\right).\end{aligned}$$

The two equations above dictate the monotone decreasing and increasing time series for degradation and synthesis, respectively.

We specify prior distributions that are the least subjective with wide variance parameters:

$$\begin{aligned}\eta_{ji0}^{(M)},\eta_{ji0}^{(H)} \sim&\mathcal{LN}(0,100^2)\quad\text{for}\quad j=0,\dots,N\\ \kappa_{i\ell}^s \sim&\mathcal{LN}(0,1)\quad\text{for}\quad \ell=0,\dots,|\boldsymbol{C}_i^{(s)}|\\ \kappa_{i\ell}^d \sim&\mathcal{LN}(0,1)\quad\text{for}\quad \ell=0,\dots,|\boldsymbol{C}_i^{(d)}|\\ (\tau_i^{(M)})^{-2} \sim&\mathcal{G}(a_M,b_M)\\ (\tau_i^{(H)})^{-2} \sim&\mathcal{G}(a_H,b_H)\end{aligned}$$

for fixed change point sets $\boldsymbol{C}_i^{(s)}$,$\boldsymbol{C}_i^{(d)}$ for all $i$, where $\mathcal{N}$, $\mathcal{LN}$, $\mathcal{G}$ denote normal, log-normal, and gamma distributions, respectively. We also assume that the change point set $\boldsymbol{C}_i^{(s)}$ has the following prior:

$$\pi(\boldsymbol{C}_i^{(s)}) \propto\varphi^{|\boldsymbol{C}_i^{(s)}|}(1-\varphi)^{T-1-|\boldsymbol{C}_i^{(s)}|}.$$

The priors are set similarly for the change point set for degradation parameter $\boldsymbol{C}_i^{(d)}$.

### 4.2 MCMC sampler

The model parameters are updated in the following order:

$$\{\eta_{ji0}\}_{j=0}^{N}\to\tau_i^2\to\{\kappa'_{it}\}_{t=0}^{T-1}\to\boldsymbol{C}_i$$

for all $i$. This whole cycle is repeated for 1,000 iterations for burn-in period and $M=10,000$ iterations for the main iteration with thinning of 10 samples, in both simulation and data analysis sections that follow. We use hat and tilde symbols to denote current and proposal values respectively.

1. We first start with $\eta_{ji0}$. We run the random walk Metropolis algorithm chain in the log-space by generating the proposal $log(\tilde{\eta}_{ji0})$ from $\mathcal{N}(log(\hat{\eta}_{ji0}),1)$ or equivalently $\tilde{\eta}_{ji0}$ from

$\hat{\eta}_{ji0}\exp(\mathcal{N}(0,1))$. Running the chain in the log-space alleviate the need to tune for step sizes for each $\eta_{ji0}$. Since this parameter is involved in the mean values at all time points, the likelihood has to be evaluated at all time points for updating each of these parameters. Similarly for $\eta_{ji0}^{(H)}$.

2. Next, we draw the variance parameter $(\tau_i^{(M)})^2$ by Gibbs sampling from inverse gamma distribution $\mathcal{IG}(a_m + N(T+1)/2, b_m + \sum_{j,t}(y_{jit}^{(M)} - \eta_{jit}^{(M)})^2/2)$.
Similarly for $(\tau_i^{(H)})^2$.

3. Next, we draw $\{\kappa_{i\ell}^s\}$ for $\ell = 0, \ldots, |\boldsymbol{C}_i^{(s)}|$ under the fixed $\boldsymbol{C}_i^{(s)}$ for each protein $i$. Again we run the chain in the log-space as we have done for $\eta_{ji0}$. i.e. draw a proposal value $\tilde{\kappa}_{i\ell}^s$ from $\hat{\kappa}_{i\ell}^s\exp(\mathcal{N}(0,1))$ and accept or reject afterwards.
Similarly for $\{\kappa_{i\ell}^d\}$.

4. Finally, we update the change point set $\boldsymbol{C}_i^{(s)}$. There are two different moves: birth of a new change point and removal (death) of an existing change point. Since these two moves are reversible in notation, we just describe the birth move here. Suppose that $\hat{\kappa}_{i\ell}^s$ covers a time period $(h_t, h_{t+m})$ that contains at least one observation time(s). Then we propose a birth of a new change point $h^* \in \{h_{t+1}, \ldots, h_{t+m-1}\}$ within the interval (chosen from one of the intermediate time points) and break the current rate parameter into two daughter parameters, namely $(\tilde{\kappa}_{i\ell}^s, \tilde{\kappa}_{i,\ell+1}^s)$ where it is required to meet

$$(h^* - h_t)\cdot\log(\tilde{\kappa}_{i\ell}^s) + (h_{t+m} - h^*)\cdot\log(\tilde{\kappa}_{i,\ell+1}^s) = (h_{t+m} - h_t)\cdot\log(\hat{\kappa}_{i\ell}^s)$$

with a random perturbation such that

$$\tilde{\kappa}_{i,\ell+1}^s = \frac{1-u}{u}\tilde{\kappa}_{i\ell}^s,$$

with $u \sim \text{Uniform}(0,1)$. Under this transformation, the Jacobian is $\frac{(\tilde{\kappa}_{i\ell}^s + \tilde{\kappa}_{i,\ell+1}^s)^2}{\hat{\kappa}_{i\ell}^s}$ for $(\hat{\kappa}_{i\ell}^s, u) \to (\tilde{\kappa}_{i\ell}^s, \tilde{\kappa}_{i,\ell+1}^s)$. Hence the Metropolis-Hastings ratio for the birth move just equals the posterior ratio times the Jacobian since the acceptance probability of this proposal is

$$\min\{1, \text{likelihood ratio} \times \text{prior ratio} \times \text{proposal ratio} \times \text{Jacobian}\}.$$

Then the Metropolis-Hastings ratio becomes

$$\left[\prod_{j,t} \frac{\exp\left\{-\frac{1}{2(\tau_i^{(M)})^2}(\ln(y_{jit}^{(M)}) - \ln(\tilde{\eta}_{jit}^{(M)}))^2\right\}\exp\left\{-\frac{1}{2(\tau_i^{(H)})^2}(\ln(y_{jit}^{(H)}) - \ln(\tilde{\eta}_{jit}^{(H)}))^2\right\}}{\exp\left\{-\frac{1}{2(\tau_i^{(M)})^2}(\ln(y_{jit}^{(M)}) - \ln(\hat{\eta}_{jit}^{(M)}))^2\right\}\exp\left\{-\frac{1}{2(\tau_i^{(H)})^2}(\ln(y_{jit}^{(H)}) - \ln(\hat{\eta}_{jit}^{(H)}))^2\right\}}\right]$$
$$\times \frac{\pi(\tilde{\kappa}_{il}^s)\pi(\tilde{\kappa}_{i,l+1}^s)}{\pi(\hat{\kappa}_{il}^s)}\frac{\varphi}{1-\varphi}$$
$$\times \frac{(\tilde{\kappa}_{i\ell}^s + \tilde{\kappa}_{i,\ell+1}^s)^2}{\hat{\kappa}_{i\ell}^s}.$$

# 5 PECA-R: Inferring synthesis and degradation rates from proteomic data not derived from a pulse-labeling experiment

PECA-R aims to estimate synthesis and degradation rates separately from proteomic concentration data (along with mRNA). For example, the data might arise from label-free experiments or proteins that were tagged with mass labels, such as TMT. The model expresses the total concentration change as a sum of increase in concentration due to new synthesis and decreased due to degradation. Note that if pulse-labeling, e.g. pulsed-SILAC, data is available, we highly recommend using PECA-pS instead of PECA-R.

In PECA-R, the synthesis and degradation rate parameters are estimated under the following assumptions:

- When the total concentration increases, it is due to the increase in the synthesis rate unless the mRNA concentration increased drastically and can explain the protein concentration increase even with an unchanged synthesis rate;
- When the total protein concentration decreases, it is due to the increase in the degradation rate unless the mRNA concentration dropped dramatically and can explain the decrease in protein concentration given constant synthesis and degradation rates.

The reason for imposing the aforementioned assumptions on the parameter space is straightforward. In label-free or TMT data, we only observe total protein changes, without separate abundance measurements for newly synthesized and existing proteins. Hence when the protein concentration changes, this model has to make a decision as to whether the synthesis rate and/or the degradation rate changed, considering the changes in mRNA concentration.

Since the total protein concentration changes can be explained by infinitely many combinations of the two rate parameters, the statistical significance score (CPS) is often more diluted in PECA-R than those values from PECA-pS. However, the PECA-pS model is not applicable unless pulse labelled samples are available, and PECA-R is the next best option for non-pulse labelled data within the PECAplus package if the estimation of synthesis and degradation is the ultimate aim of the analysis.

Note that estimating rates of synthesis and degradation (which is done with PECA-pS and PECA-R) is a goal different from simply extracting significant change points (which is done with PECA Core and PECA-N). Estimating rates aims at estimating relative ?speeds? of synthesis and degradation - they are not absolute, i.e. only applicable when comparing across genes, and have to be used with care. In contrast, significance analysis by PECA Core provides significance scores (CPS) and false discovery rates and information on the direction of the regulation (up or down) for each gene and each time point without providing rates. While PECA-R also provides CPS values, they are not as reliable for estimating significance of change as those from PECA Core.

Suppose that we have parallel mRNA and protein expression data $\boldsymbol{X} = \{x_{jit}\}$, $\boldsymbol{Y} = \{y_{jit}\}$ for protein $i = 1, \ldots, I$ in replicates $j = 1, \ldots, N$ observed over time points $(h_0, \ldots, h_T)$. Time $h_0$ indicates the time point before the samples are treated or the baseline of subsequent time points. We assume that the protein expression measurements follow log normal distributions

$$y_{jit} \sim \mathcal{LN}\left(\ln(\eta_{jit}), \tau_i^2\right)$$

after proper normalization of the data. Our goal is to infer the protein synthesis rate $\kappa_{it}^s$ and the degradation rate $\kappa_{it}^d$ during the interval $(h_t, h_{t+1})$ of length $\Delta h_t = (h_{t+1} - h_t)$ for protein $i$.

More importantly, the mean parameters are related between adjacent time points as follows:

$$\eta_{ji,t+1} = \eta_{jit} + \Delta h_t \left( f(x_{jit})\kappa^s_{it} - \eta_{jit}\kappa^d_{it} \right) \tag{2}$$

where

$$f(x) = \log(1+x)$$

for $t = 0, 1, \ldots, T-1$.

Similar to PECA-pS, we again formulated another change point model to describe the probability distribution of synthesis rates $\boldsymbol{\kappa}^s_i = (\kappa^s_{i0}, \cdots, \kappa^s_{i,T-1})$ as follows. For gene $i$, let $\boldsymbol{C}^{(s)}_i$ and $|\boldsymbol{C}^{(s)}_i|$ denote the change point sets for synthesis rates $\{t : \kappa^s_{i,t-1} \neq \kappa^s_{it} | 0, 1, \cdots, T-1\}$ and the size of the set, respectively. If the synthesis rate $\boldsymbol{\kappa}^s_i$ remained constant across time, $\boldsymbol{C}^{(s)}_i$ is an empty set; if some elements of $\boldsymbol{\kappa}^s_i$ were distinct from others, $\boldsymbol{C}^{(s)}_i$ is the set of all intermediate time points from 1 to $T-1$ with different adjacent rates. The change point set $\boldsymbol{C}^{(d)}_i$ for the degradation rate parameters $\boldsymbol{\kappa}^d_i$ are defined similarly. Again, the CPS scores are computed as the posterior probability of change point, i.e. $P(\kappa^s_{i,t-1} \neq \kappa^s_{it} | \mathbf{x}, \mathbf{y})$ for the synthesis parameter and $P(\kappa^d_{i,t-1} \neq \kappa^d_{it} | \mathbf{x}, \mathbf{y})$ for the degradation parameter of each gene $i$ at time $t$.

### 5.1 Likelihood and prior distributions

First, the likelihood of the entire model is

$$(\text{likelihood}) = \prod_{i=1}^{I} \prod_{j=1}^{N} \prod_{t=0}^{T} \frac{1}{y_{jit}\tau_i\sqrt{2\pi}} \exp\left[ -\frac{1}{2(\tau_i)^2} (\ln(y_{jit}) - \ln(\eta_{jit}))^2 \right]$$

where

$$\eta_{jit} = \eta_{ji0} + \sum_{\ell=0}^{t-1} \Delta h_\ell \left( f(x_{ji\ell})\kappa^s_{i\ell} - \eta_{ji\ell}\kappa^d_{i\ell} \right)$$

We specify prior distributions that are the least subjective with wide variance parameters:

$$\begin{aligned} \eta_{ji0} &\sim \mathcal{LN}(0, 100^2) \quad \text{for} \;\; j = 0, \ldots, N \\ \kappa^s_{i\ell} &\sim \mathcal{LN}(0, 1) \quad \text{for} \;\; \ell = 0, \ldots, |\boldsymbol{C}^{(s)}_i| \\ \kappa^d_{i\ell} &\sim \mathcal{LN}(0, 1) \quad \text{for} \;\; \ell = 0, \ldots, |\boldsymbol{C}^{(d)}_i| \\ (\tau_i)^{-2} &\sim \mathcal{G}(a, b) \end{aligned}$$

for fixed change point sets $\boldsymbol{C}^{(s)}_i$,$\boldsymbol{C}^{(d)}_i$ for all $i$ (genes), where $\mathcal{N}$, $\mathcal{LN}$, $\mathcal{G}$ denote normal, log-normal, and gamma distributions respectively. We also assume that the change point set $\boldsymbol{C}^{(s)}_i$ has the following prior:

$$\pi(\boldsymbol{C}^{(s)}_i) \propto \varphi^{|\boldsymbol{C}^{(s)}_i|} (1-\varphi)^{T-1-|\boldsymbol{C}^{(s)}_i|}$$

The priors are set similarly for the change point set for degradation parameter $\boldsymbol{C}^{(d)}_i$.

### 5.2 MCMC sampler

The model parameters are updated in the following order:

$$\{\eta_{ji0}\}_{j=0}^{N} \to \tau_i^2 \to \{\kappa'_{it}\}_{t=0}^{T-1} \to \boldsymbol{C}_i$$

for all $i$. This whole cycle is repeated for 1,000 iterations for burn-in period and $M = 10,000$ iterations for the main iteration with thinning of 10 samples, in both simulation and data analysis sections that follow. We use hat and tilde symbols to denote current and proposal values respectively.

1. We first start with $\eta_{ji0}$. We run the random walk Metropolis algorithm chain in the log-space by generating the proposal $log(\tilde{\eta}_{ji0})$ from $\mathcal{N}(log(\hat{\eta}_{ji0}), 1)$ or equivalently $\tilde{\eta}_{ji0}$ from $\hat{\eta}_{ji0}\exp(\mathcal{N}(0,1))$. Running the chain in the log-space alleviate the need to tune for step sizes for each $\eta_{ji0}$. Since this parameter is involved in the expected expression values at all time points, the likelihood has to be evaluated at all time points for updating each of these parameters.

2. Next, we draw the variance parameter $(\tau_i)^2$ by Gibbs sampling from inverse gamma distribution $\mathcal{IG}(a_m + N(T+1)/2, b_m + \sum_{j,t}(y_{jit} - \eta_{jit})^2/2)$.

3. Next, we draw $\{\kappa_{i\ell}^s\}$ for $\ell = 0, \dots, |\boldsymbol{C}_i^{(s)}|$ under the fixed $\boldsymbol{C}_i^{(s)}$ for each protein $i$. Again we run the chain in the log-space as we have done for $\eta_{ji0}$. i.e. draw a proposal value $\tilde{\kappa}_{i\ell}^s$ from $\hat{\kappa}_{i\ell}^s \exp(\mathcal{N}(0,1))$ and accept or reject afterwards.
   Similarly for $\{\kappa_{i\ell}^d\}$.

4. Finally, we update the change point set $\boldsymbol{C}_i^{(s)}$. There are two different moves: birth of a new change point and removal (death) of an existing change point. Since these two moves are reversible in notation, we just describe the birth move here. Suppose that $\hat{\kappa}_{i\ell}^s$ covers a time period $(h_t, h_{t+m})$ that contains at least one observation time(s). Then we propose a birth of a new change point $h^* \in \{h_{t+1}, \dots, h_{t+m-1}\}$ within the interval (chosen from one of the intermediate time points) and break the current rate parameter into two daughter parameters, namely $(\tilde{\kappa}_{i\ell}^s, \tilde{\kappa}_{i,\ell+1}^s)$ where it is required to meet

   $$(h^* - h_t) \cdot \log(\tilde{\kappa}_{i\ell}^s) + (h_{t+m} - h^*) \cdot \log(\tilde{\kappa}_{i,\ell+1}^s) = (h_{t+m} - h_t) \cdot \log(\hat{\kappa}_{i\ell}^s)$$

   with a random perturbation such that

   $$\tilde{\kappa}_{i,\ell+1}^s = \frac{1-u}{u}\tilde{\kappa}_{i\ell}^s,$$

   with $u \sim \text{Uniform}(0,1)$. Under this transformation, the Jacobian is $\frac{(\tilde{\kappa}_{i\ell}^s + \tilde{\kappa}_{i,\ell+1}^s)^2}{\hat{\kappa}_{i\ell}^s}$ for $(\hat{\kappa}_{i\ell}^s, u) \to (\tilde{\kappa}_{i\ell}^s, \tilde{\kappa}_{i,\ell+1}^s)$. Hence the Metropolis-Hastings ratio for the birth move just equals the posterior ratio times the Jacobian since the acceptance probability of this proposal is

   $$\min\{1, \text{likelihood ratio} \times \text{prior ratio} \times \text{proposal ratio} \times \text{Jacobian}\}.$$

Then the Metropolis-Hastings ratio becomes

$$\left[\prod_{j,t}\frac{\exp\left\{-\frac{1}{2(\tau_i)^2}(\ln(y_{jit})-\ln(\tilde{\eta}_{jit}))^2\right\}}{\exp\left\{-\frac{1}{2(\tau_i)^2}(\ln(y_{jit})-\ln(\hat{\eta}_{jit}))^2\right\}}\right]$$
$$\times\frac{\pi(\tilde{\kappa}^s_{il})\pi(\tilde{\kappa}^s_{i,l+1})}{\pi(\hat{\kappa}^s_{il})}\frac{\varphi}{1-\varphi}$$
$$\times\frac{(\tilde{\kappa}^s_{i\ell}+\tilde{\kappa}^s_{i,\ell+1})^2}{\hat{\kappa}^s_{i\ell}}.$$

# References


[1] M. Ashburner, C. A. Ball, J. A. Blake, D. Botstein, H. Butler, J. M. Cherry, A. P. Davis, K. Dolinski, S. S. Dwight, J. T. Eppig, M. A. Harris, D. P. Hill, L. Issel-Tarver, A. Kasarskis, S. Lewis, J. C. Matese, J. E. Richardson, M. Ringwald, G. M. Rubin, and G. Sherlock. Gene Ontology: tool for the unification of biology. *Nature Genetics*, 25(1):25–29, May 2000.

[2] J. Besag. On the statistical analysis of dirty pictures. *Journal of the Royal Statistical Society. Series B (Methodological)*, 48(3):259–302, 1986.

[3] P. J. Green. Reversible jump Markov chain Monte Carlo computation and Bayesian model determination. *Biometrika*, 82(4):711–732, 1995.

[4] L. J. Jensen, M. Kuhn, M. Stark, S. Chaffron, C. Creevey, J. Muller, T. Doerks, P. Julien, A. Roth, M. Simonovic, P. Bork, and C. von Mering. STRING 8—-a global view on proteins and their functional interactions in 630 organisms. *Nucleic Acids Research*, 37(suppl_1):D412–D416, 2009.

[5] C. E. Rasmussen and C. K. I. Williams. *Gaussian Processes for Machine Learning (Adaptive Computation and Machine Learning)*. The MIT Press, 2005.

[6] G. Teo, C. Vogel, D. Ghosh, S. Kim, and H. Choi. PECA: A novel statistical tool for deconvoluting time-dependent gene expression regulation. *Journal of Proteome Research*, 13(1):29–37, 2014. PMID: 24229407.

[7] Z. Wei and H. Li. A markov random field model for network-based analysis of genomic data. *Bioinformatics*, 23(12):1537–1544, 2007.
